# SEDLIN Forms Homodimers: Characterisation of SEDLIN Mutations and Their Interactions with Transcription Factors MBP1, PITX1 and SF1

**DOI:** 10.1371/journal.pone.0010646

**Published:** 2010-05-14

**Authors:** Jeshmi Jeyabalan, M. Andrew Nesbit, Juris Galvanovskis, Richard Callaghan, Patrik Rorsman, Rajesh V. Thakker

**Affiliations:** 1 Academic Endocrine Unit, Nuffield Department of Clinical Medicine, Oxford Centre for Diabetes, Endocrinology and Metabolism, University of Oxford, Churchill Hospital, Oxford, United Kingdom; 2 Diabetes Research Laboratories, Nuffield Department of Clinical Medicine, Oxford Centre for Diabetes, Endocrinology and Metabolism, University of Oxford, Churchill Hospital, Oxford, United Kingdom; 3 Nuffield Department of Clinical Laboratory Sciences, University of Oxford, John Radcliffe Hospital, Oxford, United Kingdom; University of Pittsburgh, United States of America

## Abstract

**Background:**

SEDLIN, a 140 amino acid subunit of the Transport Protein Particle (TRAPP) complex, is ubiquitously expressed and interacts with the transcription factors *c-myc* promoter-binding protein 1 (MBP1), pituitary homeobox 1 (PITX1) and steroidogenic factor 1 (SF1). SEDLIN mutations cause X-linked spondyloepiphyseal dysplasia tarda (SEDT).

**Methodology/Principal Findings:**

We investigated the effects of 4 missense (Asp47Tyr, Ser73Leu, Phe83Ser and Val130Asp) and the most C-terminal nonsense (Gln131Stop) SEDT-associated mutations on interactions with MBP1, PITX1 and SF1 by expression in COS7 cells. Wild-type SEDLIN was present in the cytoplasm and nucleus and interacted with MBP1, PITX1 and SF1; the SEDLIN mutations did not alter these subcellular localizations or the interactions. However, SEDLIN was found to homodimerize, and the formation of dimers between wild-type and mutant SEDLIN would mask a loss in these interactions. A mammalian SEDLIN null cell-line is not available, and the interactions between SEDLIN and the transcription factors were therefore investigated in yeast, which does not endogenously express SEDLIN. This revealed that all the SEDT mutations, except Asp47Tyr, lead to a loss of interaction with MBP1, PITX1 and SF1. Three-dimensional modelling studies of SEDLIN revealed that Asp47 resides on the surface whereas all the other mutant residues lie within the hydrophobic core of the protein, and hence are likely to affect the correct folding of SEDLIN and thereby disrupt protein-protein interactions.

**Conclusions/Significance:**

Our studies demonstrate that SEDLIN is present in the nucleus, forms homodimers and that SEDT-associated mutations cause a loss of interaction with the transcription factors MBP1, PITX1 and SF1.

## Introduction

**Figure 1:**
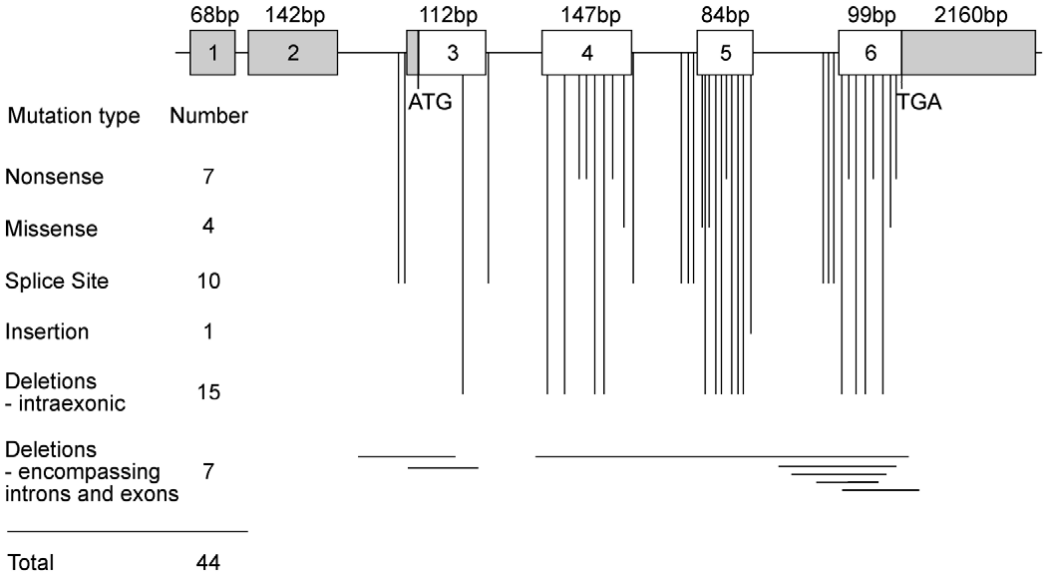
Schematic representation of genomic organization of the SEDL gene, illustrating the 44 identified mutations. The human *SEDL* gene consists of six exons that span approximately 22 Kb of genomic DNA and encode a 140 amino acid protein. The 420bp coding region (open boxes) is organised into 4 exons (exon 3 to exon 6) and 3 introns (indicated by a line, not to scale). Non-coding exons (filled boxes) consist of exons 1 and 2, the 5′ portion of exon 3 and the 3′ portion of exon 6. The sizes of the exons, and the translation Start (ATG) and Stop (TGA) codons in exon 3 and 6, respectively, are indicated. The locations of the 44 mutations are indicated, and these consist of: 7 nonsense, 4 missense, 10 splice site, 1 insertion, 15 intraexonic deletions, and 7 deletions that encompassed introns and exons. The four missense mutations (Asp47Tyr, Ser73Leu, Phe83Ser and Val130Asp) and one nonsense mutation (Gln131Stop) were selected for further functional studies (Figures 3, 4, 6 and 7).

**Figure 2:**
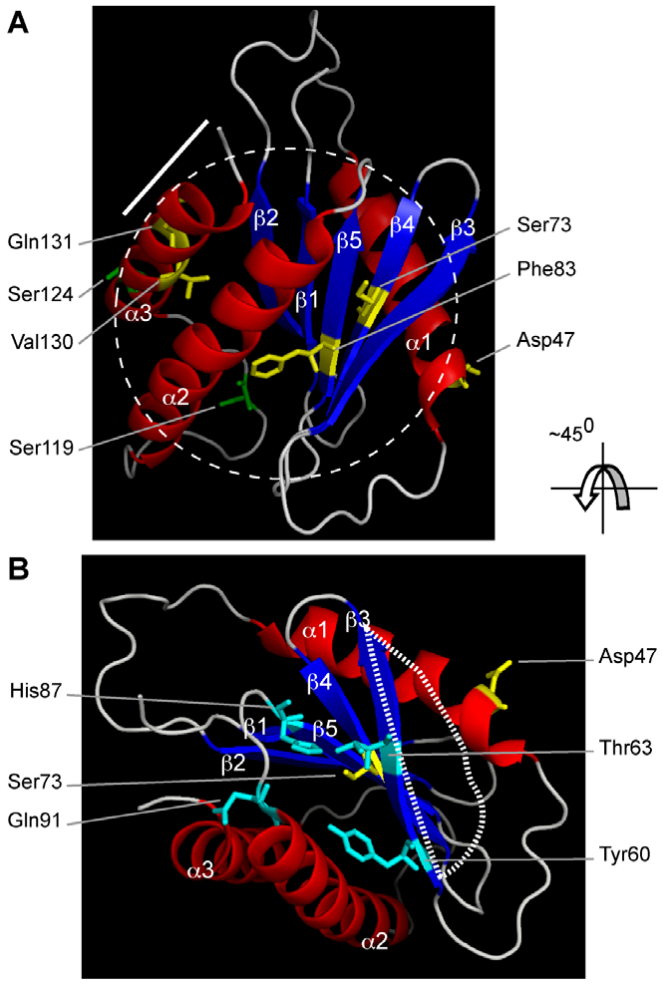
Three-dimensional model of SEDLIN showing locations of residues involved in the mutations studied. The model is based on a published model of mouse Sedlin [2]. (A) A ribbon model of Sedlin showing the alpha helices in red, beta strands in blue and the residues involved in the missense mutations (Asp47Tyr, Ser73Leu, Phe83Ser, and Val130Asp) in yellow. The bold line indicates the 10 C-terminal amino acids that would be deleted by the nonsense SEDLIN mutation (Gln131Stop) shown in yellow. The dashed circle indicates the hydrophobic core of Sedlin. The Ser119 and Ser124 residues that were predicted to be phosphorylation sites (NetPhos2.0, MotifScan and ELM databases) are indicated in green. (B) An ∼45° rotated view of the ribbon model of Sedlin showing the SEDT-associated mutated residues Asp47 and Ser73 (yellow), in SEDT, which together with the residues Tyr60, Thr63, His67 and Gln91 (light blue) aid in forming the hydrophobic groove (indicated by dotted bar line). The alpha helices are shown in red, beta strands in blue, and the linker region in grey.

SEDLIN, which is one of the subunits of the Transport Protein Particle (TRAPP) complex, is involved in the targeting and fusion of endoplasmic reticulum (ER)-derived transport vesicles to the Golgi acceptor compartment [1]. SEDLIN, which is encoded by the spondyloepiphyseal dysplasia late (*SEDL*) gene (Figure 1) is a highly conserved 140 amino acid protein with a >95% identity amongst vertebrate orthologues and a >30% identity to the yeast Trs20p [2]. The crystal structure of mouse Sedlin shows it to have a single domain structure (Figure 2) that contains 20 solvent-accessible apolar residues [2]. Four of these apolar residues constitute the hydrophobic pocket (Pro16) and the hydrophobic groove (Phe40, Leu48 and Phe67), both of which may act as protein-binding sites [2]. Indeed, SEDLIN has been reported to interact with proteins that are not part of the TRAPP complex, but are either transcription factors, such as the *c-myc* promoter-binding protein 1 (MBP1), pituitary homeobox 1 (PITX1) and steroidogenic factor 1 (SF1) [3], [4], or the intracellular chloride channels, CLIC1 and CLIC2 [5]. Interestingly, SEDLIN has been reported to localize to the perinuclear structures [6], although the reported interactions with the transcription factors MBP1, PITX1 and SF1, would also suggest a role for SEDLIN in the nucleus. Mutations of SEDLIN have been reported in patients with spondyloepiphyseal dysplasia tarda (SEDT), an X-linked osteochondrodysplasia that is characterised by short stature, a disproportionate short trunk, barrel-shaped chest, narrowing of the intervertebral disc spaces, platyspondyly, a shortened femoral neck, and early onset secondary osteoarthritis which may require hip replacement before the age of 40 years [7]. The forty-four disease-causing *SEDL* mutations, which had been reported at the commencement of these studies, consisted of 40 that resulted in premature truncations and 4 that were missense mutations (Asp47Tyr, Ser73Leu, Phe83Ser and Val130Asp) (Figure 1). However, the functional consequences of these *SEDL* mutations at the cellular level remain unidentified and we hypothesised that these may involve a loss of interactions with MBP1, PITX1 and SF1, particularly as PITX1 and MBP1, which is also referred to as enolase1, have been reported to have roles in endochondral ossification and maintenance of adult bone [8], [9]. Moreover, PITX1 expression has been reported to be significantly reduced in osteoarthritic cartilage [9], and citrullinated enolase1, which is a known rheumatoid arthritis autoantigen, is expressed at high levels in rheumatoid joints [8]. We therefore undertook studies that aimed to explore the nuclear expression of SEDLIN, which would be consistent with its interactions with the transcription factors MBP1, PITX1 and SF1, and to determine the functional consequences of disease-associated SEDLIN mutations on the subcellular localization and interactions with PITX1, MBP1 and SF1. We focused on the 4 SEDT-associated missense mutations (Figure 1) as these were predicted to yield a full-length protein and were not likely to affect the tertiary structure of SEDLIN substantially, as well as the most C-terminal nonsense (Gln131Stop) mutation, which if translated, is predicted to result in the loss of the last ten amino acids.

## Results

### Subcellular Localization of SEDLIN

**Figure 3:**
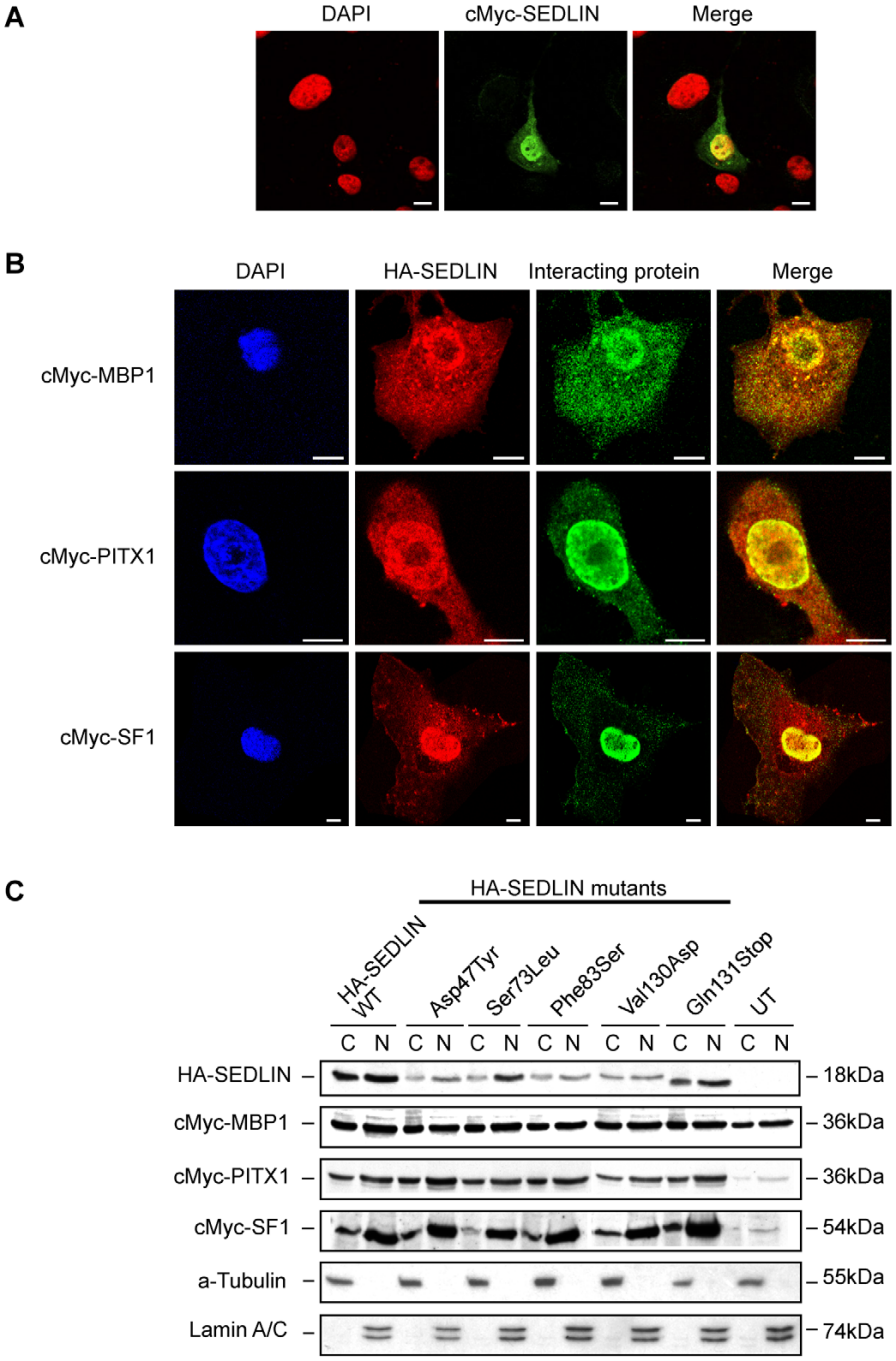
Subcellular co-localization of wild-type and mutant SEDLINs, MBP1, PITX1 and SF1. (A) COS7 cells were transfected with wild-type or mutant (Asp47Tyr, Ser73Leu, Phe83Ser, Val130Asp and Gln131Stop) cMyc-SEDLIN constructs and visualised by immunofluorescence. The wild-type cMyc-SEDLIN (green) and mutant forms (data not shown) were found to localize to the nucleus and cytoplasm. DAPI, which stains nuclei (shown as red), colocalized with SEDLIN (yellow in merged image). (B) Wild-type or mutant HA-SEDLINs (red) were co-transfected with cMyc-MBP1, cMyc-PITX1 or cMyc-SF1 constructs and visualized by immunofluorescence (green). Wild-type SEDLIN and mutant SEDLINs (data not shown) co-localized (yellow) to the nucleus with the three transcription factors and to punctate structures within the cytoplasm with MBP1. (C) Western blot analysis of subcellular fractions (N - nuclear, C–cytoplasmic) from COS7 cells transiently co-transfected with wild-type (WT) or one of the 5 mutant HA-SEDLIN constructs, and cMyc-MBP1, cMyc-PITX1 or cMyc-SF1 constructs. Use of anti-HA antibody detected the expected 18 kDa wild-type and mutant SEDLIN proteins, and anti-cMyc antibody detected the expected 36 kDa, 36 kDa and 54 kDa MBP1, PITX1 and SF1 proteins, respectively, which were seen in the nuclear and cytoplasmic fractions, thereby confirming the immunofluorescence results. Western blots with anti-α-Tubulin and anti-Lamin A/C antibodies confirmed that the nuclear and cytoplasmic fractions were free from detectable amounts of cytoplasmic and nuclear fractions, respectively. Untransfected (UT) cells-not transfected with HA-SEDLIN. Wild-type and mutant SEDLINs were found in the nuclear and cytoplasmic fractions, and the SEDLIN mutants did not lead to an altered subcellular localization of MBP1, PITX1 and SF1. Scale bars, 10 µm.

SEDLIN has been postulated to play a role in ER-Golgi vesicular transport and has been reported to localize to perinuclear structures [6]. However, its interaction with the transcription factors MBP1, PITX1 and SF1 suggested a possible nuclear localization. We explored this possibility by confocal immunofluorescence studies of COS7 cells that were transiently transfected with cMyc- or HA-tagged constructs of wild-type (WT) SEDLIN, mutant SEDLIN, wild-type MBP1, PITX1 or SF1. We specifically chose to generate the tagged constructs using cMyc- and HA-tags as these are small proteins (<1.5 kDa) [10], [11] unlike a green fluorescence protein (GFP)-tag (∼27 kDa) [12], and hence less likely to affect the biochemical and biophysical properties of SEDLIN, which is itself only 16 kDa in size. Wild-type cMyc-SEDLIN expression was observed in the nucleus and cytoplasm (Figure 3A), and the SEDT-associated mutations (Asp47Tyr, Ser73Leu, Phe83Ser, Val130Asp and Gln131Stop) were found to have a similar cytoplasmic and nuclear localization, and not to affect this subcellular localization (data not shown). MBP1, PITX1 and SF1 were also expressed in the nucleus and cytoplasm, but PITX1 and SF1 co-localized with SEDLIN in the nucleus only, whereas MBP1 co-localized with SEDLIN in the nucleus and cytoplasm (Figure 3B). These results were confirmed by Western blot analysis of subcellular fractions, which demonstrated the presence of wild-type and mutant SEDLINs in both the cytoplasmic and nuclear fractions (Figure 3C). This presence of wild-type and mutant SEDLINs with the transcription factors MBP1, PITX1 and SF1 in the nucleus and cytoplasm (Figures 3B and C) suggested that the effects of the SEDT-associated mutations on the interactions between SEDLIN and these transcription factors may occur in either compartment, and these effects were therefore assessed for in total cell lysates.

### Interaction of wild-type and mutant SEDLINs with MBP1, PITX1 and SF1 is masked by formation of SEDLIN dimers in mammalian cells

**Figure 4:**
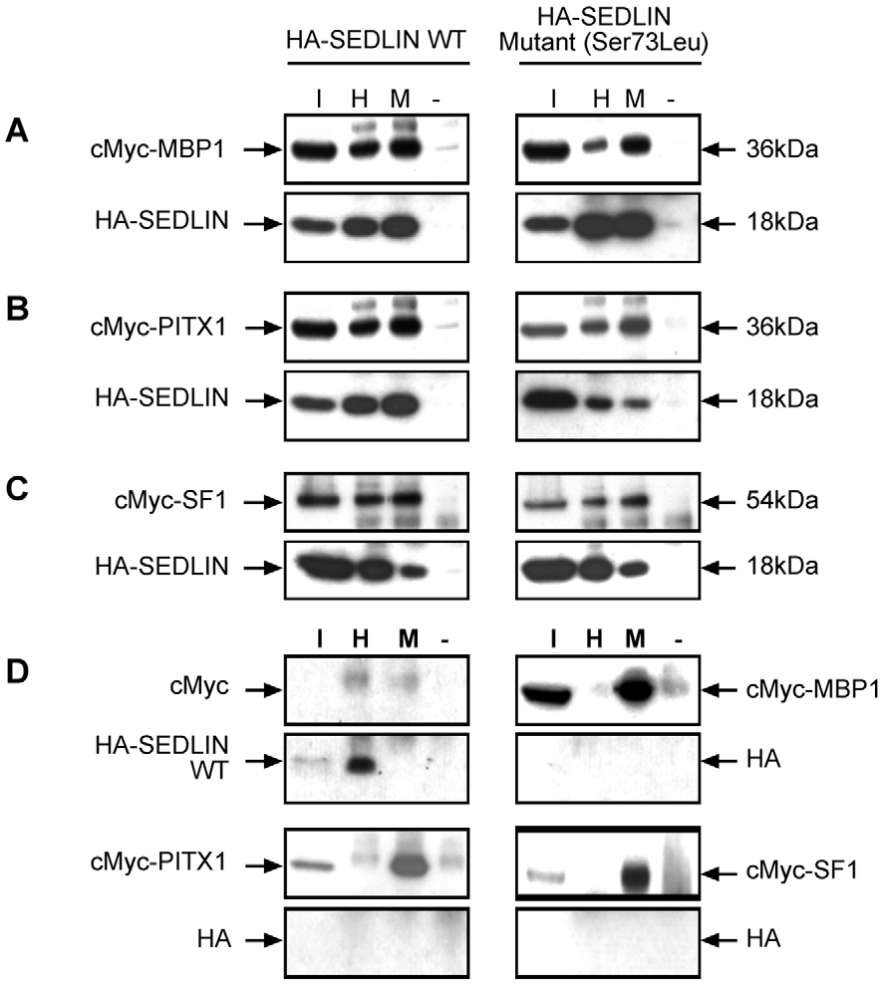
Interactions between SEDLIN, and MBP1, PITX1 and SF1. Co-immunoprecipitation studies using COS7 cells demonstrated interactions between wild-type SEDLIN, mutant SEDLINs (Asp47Tyr, Ser73Leu, Phe83Ser, Val130Asp and Gln131Stop, only data from Ser73Leu SEDLIN shown) and MBP1, PITX1 and SF1. (A) cMyc-MBP1 co-transfected with wild-type or mutant HA-SEDLIN; (B) cMyc-PITX1 co-transfected with wild-type or mutant HA-SEDLIN; (C) cMyc-SF1 co-transfected with wild-type or mutant HA-SEDLIN; and (D) empty cMyc vector co-transfected with HA-SEDLIN or empty HA vector co-transfected with cMyc-MBP1, cMyc-PITX1 or cMyc-SF1 constructs. Lysates were incubated with either anti-cMyc polyclonal antibody (M) or anti-HA polyclonal antibody (H), or without any antibody as a negative control (−) and immunoprecipitated with Protein G-Sepharose beads. Protein complexes were eluted and resolved on SDS-PAGE followed by Western blot analysis using an antibody to the cMyc epitope for MBP1, PITX1 and SF1, and to the HA epitope for SEDLIN. Five percent of the lysate (input (I)) that was used for the immunoprecipitation was electrophoresed in parallel with the immunoprecipitated lysates. Wild-type SEDLIN co-immunoprecipitated MBP1, PITX1 and SF1 and the SEDLIN mutations (Asp47Tyr, Ser73Leu, Phe83Ser, Val130Asp and Gln131Stop) did not disrupt these interactions (representative data for mutant Ser73Leu SEDLIN shown).

**Figure 5:**
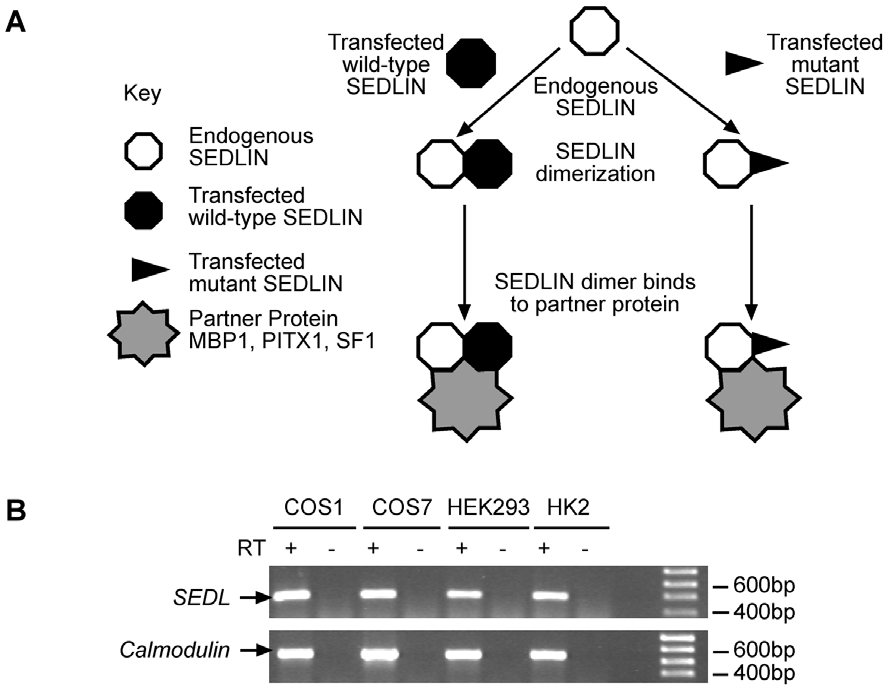
Schematic model for SEDLIN homodimerization and *SEDL* expression in common cell lines. (A) Homodimers that have formed between the transfected mutant cMyc-SEDLIN and the endogenously expressed wild-type SEDLIN could mask the loss of interaction between mutant SEDLINs and MBP1, PITX1 and SF1 in transfected COS7 cells. Thus, although the mutant SEDLIN may not directly interact with MBP1, PITX1 or SF1, the endogenously expressed SEDLIN will interact with these transcription factors, and hence the resultant homodimers will overall be seen to interact with the transcription factors. This proposed model provides an explanation for the observed results in the transfected cells. However, it is important to note that this situation of homodimers consisting of a wild-type and mutant SEDLIN would not normally occur in males affected with SEDT, as they are hemizygous and their cells would normally express the mutant SEDLIN; however this situation would occur in SEDT heterozygous carrier females because the *SEDL* gene escapes X-chromosome inactivation [13] and hence their cells would express both the wild-type and mutant SEDLINs. (B) RT-PCR analysis was used to detect the endogenous expression of *SEDL* in COS7, COS1, HEK293 and HK2 kidney cells, as a reliable SEDLIN antibody is not available. Detection of *Calmodulin* expression was used as an internal control for RNA quality and concentration; (+) with RTase, (−) without RTase.

**Figure 6:**
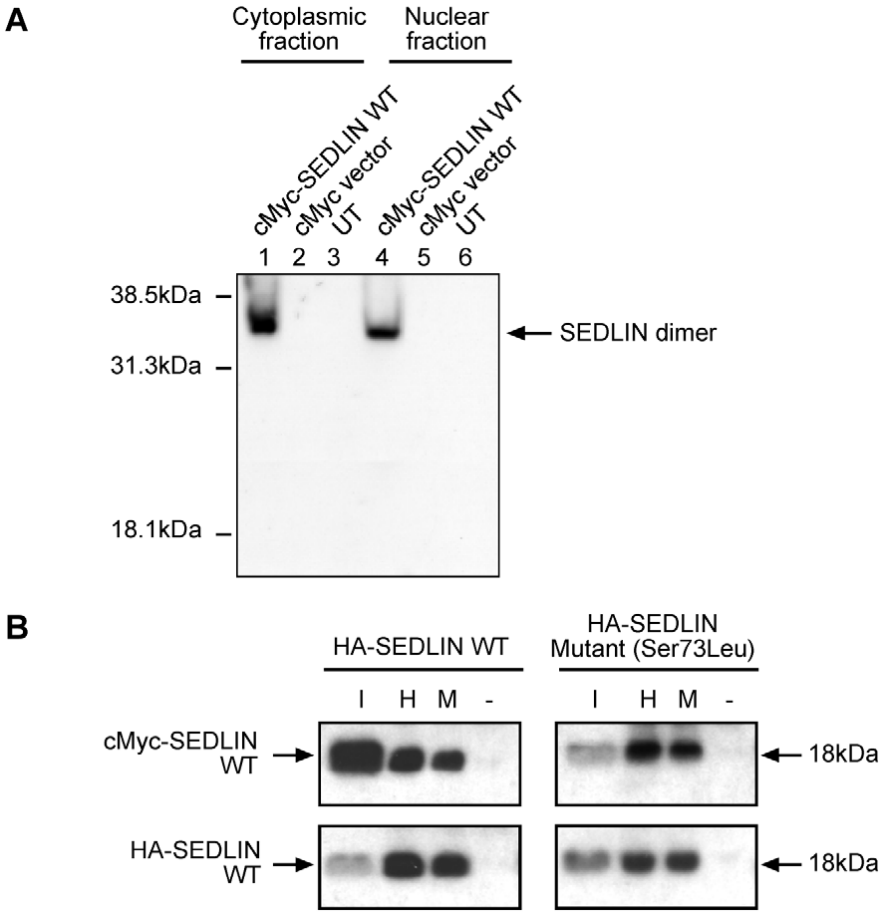
SEDLIN forms homodimers. (A) Western blot analysis, using a monoclonal anti-cMyc antibody, of cell lysates obtained from COS7 cells transfected with cMyc-SEDLIN or cMyc vector alone, and resolved by continuous non-denaturing PAGE; lanes 1-3, cytoplasmic fractions; lanes 4–6, nuclear fractions; lane 1 and 4, transfected with cMyc-SEDLIN wild-type (WT) construct; lanes 2 and 5, transfected with cMyc vector alone; and lanes 3 and 6, untransfected (UT) cells. cMyc-SEDLIN, an 18 kDa protein (Figures 3 and 4) appeared as a 36 kDa protein on non-denaturing PAGE of cytoplasmic and nuclear fractions obtained from COS7 cells transfected with the cMyc-SEDLIN wild-type (WT) construct, consistent with homodimerization of SEDLIN protein. (B) Co-immunoprecipitation of wild-type (WT) cMyc-SEDLIN with wild-type or mutant HA-SEDLINs (data for mutant Ser73Leu, shown). Anti-cMyc antibody co-immunoprecipitated wild-type HA-SEDLIN in the presence of cMyc-SEDLIN (lane M) and anti-HA antibody co-immunoprecipitated cMyc-SEDLIN in the presence of HA-SEDLIN (lane H); in the absence of the anti-cMyc or anti-HA antibodies SEDLIN was not immunoprecipitated (lane -). Five percent of the lysate (input (I)) that was used for the immunoprecipitation was electrophoresed in parallel with immunoprecipitated lysates. Similar results were observed for the other SEDLIN mutants (Asp47Tyr, Phe83Ser, Val130Asp and Gln131Stop) (data not shown).

Interactions in mammalian cells between wild-type (WT) and mutant (Asp47Tyr, Ser73Leu, Phe83Ser, Val130Asp and Gln131Stop) SEDLINs and the transcription factors MBP1, PITX1 and SF1 were investigated by co-transfecting COS7 cells with full-length wild-type SEDLIN or mutant SEDLIN constructs that were tagged with an HA epitope, and full-length wild-type MBP1, PITX1 and SF1 constructs that were tagged with a cMyc epitope. The cMyc empty vector was used as a negative control. When wild-type or mutant HA-SEDLIN was immunoprecipitated with anti-HA antibody, the immunoreactive bands of cMyc-MBP1, cMyc-PITX1 and cMyc-SF1 (Figures 4A–C) were detected in the immunoprecipitate. Wild-type and mutant HA-SEDLINs were reciprocally co-immunoprecipitated with cMyc-MBP1, cMyc-PITX1 and cMyc-SF1 using anti-cMyc antibody (Figures 4A–C). The co-immunoprecipitation of MBP1, PITX1 and SF1 with wild-type and mutant SEDLIN was specific as the HA tag alone did not co-immunoprecipitate these proteins (Figure 4D). These results demonstrate that wild-type SEDLIN interacts with MBP1, PITX1 and SF1; however, they also suggest that the SEDT-associated mutations do not affect the interactions between the mutant SEDLINs and the transcription factors MBP1, PITX1 or SF1 (Figures 4A–C). This was a surprising result, and the apparent lack of any effect on these interactions by the SEDT-associated mutations was further explored. One possibility for not observing any effects of the mutant SEDLINs on the interactions with the transcription factors in the transfected cells is that SEDLIN forms dimers (Figure 5A). Thus, the formation of dimers between the endogenously expressed wild-type SEDLIN and the transfected mutant SEDLIN would mask the loss of any interaction between the mutant SEDLIN and the transcription factor, as the endogenously expressed wild-type SEDLIN would bind to the transcription factor. COS7 cells were found to endogenously express wild-type SEDLIN, consistent with this possibility (Figure 5B) of homodimerization, which was investigated further by non-denaturing gel electrophoresis and co-immunoprecipitation (Figure 6). Western blot analysis of nuclear and cytoplasmic fractions obtained from COS7 cells transfected with wild-type cMyc-SEDLIN, and resolved by non-denaturing gel electrophoresis, revealed the presence of a 36 kDa band which is consistent with the formation of SEDLIN dimers in both compartments (Figure 6A). In addition, co-immunoprecipitation studies using Western blot analysis of lysates of COS7 cells that were co-transfected with wild-type cMyc-SEDLIN and wild-type or mutant HA-SEDLINs, demonstrated that wild-type cMyc-SEDLIN was able to co-immunoprecipitate wild-type and mutant HA-SEDLINs (Figure 6B), thereby revealing that mutant SEDLIN interacts with wild-type SEDLIN. Thus, SEDLIN forms homodimers, including dimers between wild-type and mutant SEDLINs, as postulated by our proposed model (Figure 5A) to explain the apparent lack of observable effects of SEDLIN mutations on the interactions with the transcription factors MBP1, PITX1 and SF1. However, it is important to note that affected SEDT males are hemizygous, and their cells would express only the mutant SEDLIN, and that the situation of homodimer formation between wild-type and mutant SEDLIN would only occur in carrier females as the *SEDL* gene escapes X-chromosome inactivation [13]. This indicates that to study the effects of the transfected SEDLIN mutants on interactions with MBP1, PITX1 and SF1, and simulate the situation in the hemizygous affected SEDT males, one requires a cell line that does not express endogenous wild-type SEDLIN. An assessment of 3 other mammalian cell lines (COS1, HEK293 and HK2) revealed all of them to express SEDLIN (Figure 5B), and as SEDLIN-null cell lines are not available from SEDT patients or a mouse model, we further explored the effects of mutant SEDLINs on the interaction with the transcription factors MBP1, PITX1 and SF1, in yeast cells.

### SEDLIN interactions with transcription factors MBP1, PITX1 and SF1 in yeast cells

**Figure 7:**
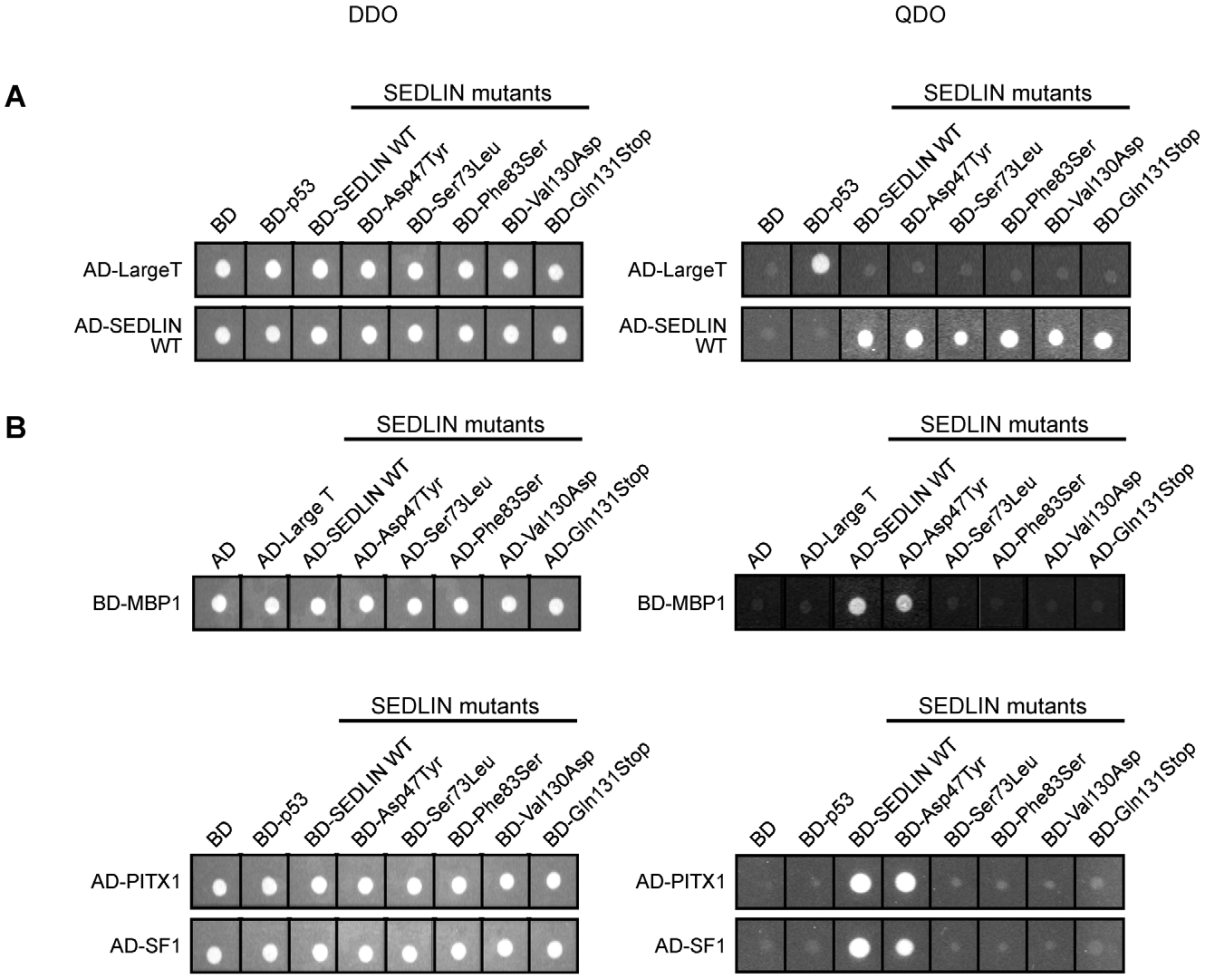
Interactions between SEDLIN, MBP1, PITX1 and SF1 using the yeast two-hybrid assay. Yeast cells, which do not have endogenous expression of SEDLIN, were used to investigate the interactions of wild-type or mutant SEDLIN (Asp47Tyr, Ser73Leu, Phe83Ser, Val130Asp and Gln131Stop) with wild-type SEDLIN, MBP1, PITX1 or SF1. The yeast reporter strain AH109 was used, and p53 and the SV40 large T antigen, which are known to interact [27], were used as a positive control. The yeasts were transformed with the vectors containing: (A) wild-type SEDLIN in pGADT7-AD (AD-WT) and either wild-type or mutant SEDLINs in pGBKT7-BD (BD-WT, BD-Asp47Tyr, BD-Ser73Leu, BD-Phe83Ser, BD-Val130Asp or BD-Gln131Stop). (B) wild-type and mutant AD-SEDLINs or BD-SEDLIN and each of the transcription factors BD-MBP1, AD-PITX1, AD-SF1. Yeast growth was monitored for 48 hrs after spotting and incubation at 30°C using either double drop out, DDO (Leu^-^Trp^-^), media as a control or quaternary drop out, QDO (Leu^-^Trp^-^Ade^-^His^-^), media in which the growth is dependent on the physical interaction between BD-SEDLIN and AD-transcription factors, or AD-SEDLIN and the BD-transcription factor. The wild-type SEDLIN interacts with wild-type SEDLIN and all of the mutant SEDLIN proteins, consistent with the proposed model for the formation of homodimers. However, the MBP1, PITX1 and SF1 fusion proteins, which interact with the wild-type SEDLIN, interacted only with the mutant Asp47Tyr SEDLIN, but not with the mutant Ser73Leu, Phe83Ser, Val130Asp and Gln131Stop SEDLINs.

Yeast cells express Trs20p, which is a SEDLIN homologue that is located in the ER and Golgi membranes [1], [14]. Trs20p has <40% identity at the amino acid level to mammalian SEDLIN [2], thereby reducing the likelihood that it may form dimers with the transfected human SEDLIN and interfere with the interaction between mutant SEDLINs and the transcription factors. We therefore used a yeast two-hybrid assay to assess for interactions between wild-type and mutant SEDLINs and the transcription factors MBP1, PITX1 and SF1. The results demonstrated that wild-type SEDLIN interacted with wild-type SEDLIN (Figure 7A), consistent with the findings of dimer formation in mammalian COS7 cells (Figure 6A). Wild-type SEDLIN was also found to interact with each of the SEDLIN mutants (Figure 7A), and hence consistent with the results of the co-immunoprecipitation studies in mammalian COS7 (Figure 6B). In addition, wild-type SEDLIN was demonstrated to interact with MBP1, PITX1 and SF1 (Figure 7B), yielding results that were similar to those obtained from the mammalian COS7 cells (Figure 4). However, the SEDLIN missense mutations Ser73Leu, Phe83Ser, Val130Asp and the nonsense mutation Gln131Stop lead to a loss of interaction with MBP1, PITX1 and SF1 (Figure 7B), although the Asp47Tyr mutation, which involves a highly conserved residue did not lead to a loss of interaction with any of these transcription factors. Co-expression of these proteins was confirmed by Western blot analysis of yeast protein extracts from each clone (data not shown). These results from a yeast two-hybrid assay demonstrate that 3 of the SEDT-associated missense mutations (Ser73Leu, Phe83Ser, Val130Asp) and the most C-terminal nonsense mutation (Gln131Stop) result in a loss of direct interaction with the transcription factors MBP1, PITX1 and SF1. In addition, these results confirm the direct interactions that lead to the formation of dimers between human wild-type SEDLIN and mutant SEDLINs.

### Structural effects of SEDLIN mutants

An analysis of the three-dimensional structure of SEDLIN revealed that the SEDLIN mutants (Ser73Leu, Phe83Ser, Val130Asp and Gln131Stop) that lead to a loss of interaction with the MBP1, PITX1 and SF1, are buried within the hydrophobic core domain of SEDLIN and these may affect the structural integrity of SEDLIN, as well as the surfaces that are involved in interactions with these proteins (Figure 2). For example, mutation of polar Ser73 to Leu73 would result in the loss of hydrogen bonding with the side chain of Tyr60 and His87, and with the backbone nitrogen atoms of Thr63 and Gln91 and the disruption of a hydrophobic groove between α1 and β3 [2]. Similarly, the Phe83 and Val130 residues are in the hydrophobic region within the interior of the protein, and mutations of these apolar residues to polar residues (serine and aspartic acid, respectively) will likely disrupt the interactions within the hydrophobic core (Figure 2), leading to a misfolding of SEDLIN that will disrupt protein-protein interactions. The loss of interactions between the truncated Gln131Stop mutant protein, which comprises >90% of the full-length 140 amino acid SEDLIN protein, and MBP1, PITX1 and SF1, also indicate that protein misfolding rather than a lack of protein is the most likely explanation for a loss of interaction with the transcription factors. The Asp47Tyr SEDLIN mutation did not lead to a loss of interaction with MBP1, PITX1 and SF1. This mutation of the Asp47 residue, which is exposed on the surface of SEDLIN, is unlikely to cause aberrant protein folding and this is a likely explanation for a lack of disruption in the interactions with MBP1, PITX1 and SF1.

## Discussion

Our results show that SEDLIN forms homodimers (Figures 6 and 7), is localized to the cytoplasm and nucleus (Figure 3), and that wild-type SEDLIN co-localizes and interacts with the transcription factors MBP1, PITX1 and SF1 (Figures 3, 4 and 7). In addition, our results show that SEDT-associated SEDLIN mutations do not result in abnormalities of subcellular localization (Figure 3), but those involving residues within the hydrophobic core (Figure 2) do lead to a loss of interactions with the transcription factors MBP1, PITX1 and SF1 (Figure 7). The relevance and further insights provided by these findings may help to elucidate the role of SEDLIN in cellular functions.

The dimerization of SEDLIN and its nuclear localization points to additional roles for mammalian SEDLIN, which with its yeast orthologue Trs20p, have previously been reported to be present as monomers in the TRAPP complex, where SEDLIN is involved in tethering vesicles [1]. However, the formation of SEDLIN dimers (Figures 6 and 7) and their nuclear localization (Figures 3 and 6) together with the interactions with MBP1, PITX1 and SF1 (Figures 4 and 7) indicate a role in modulating transcription and the situation may be analogous to that observed with the c-Jun N-terminal kinase (JNK)-interacting protein 1 (JIP-1). The monomeric form of JIP-1 is a cytoplasmic scaffold protein that is essential for the organization of the JNK signalling pathway, while the dimeric form is predominantly located in the nucleus, where it likely mediates transcription control of pathways involved in prevention of neuronal death [15]. The dimerization of JIP-1 involves post-translation modification and is dependent on phosphorylation [15], and it is interesting to note that SEDLIN has predicted phosphorylation sites at Ser119 and Ser124 (NetPhos2.0, MotifScan and ELM databases) (Figure 2A). The mechanisms that transport SEDLIN into the nucleus remain to be defined, as SEDLIN does not have a nuclear localization signal (NLS). It seems likely that SEDLIN may be co-transported with the interacting proteins that contain NLSs, thereby enabling it to enter the nucleus [16]. Within the nucleus SEDLIN, given its interaction with the transcription factors MBP1, PITX1 and SF1, may act as either a co-repressor or co-activator of gene transcription. For example, SEDLIN acts as a repressor molecule by binding to PITX1 and SF1 and inhibiting transactivation [4], as well as an activator by binding to repressor molecules such as MBP1 that facilitate gene transcription [3]. The roles of these interactions between SEDLIN and MBP1, PITX1 and SF1 in skeletal biology remain to be elucidated. However, *in vitro* studies have shown that SEDLIN inhibits MBP1 mediated repression of *c-myc* transcription [3], and MBP1 has been reported to be associated with two different cellular processes during endochondral ossification; cell proliferation in the proliferative and upper hypertrophic layers, and apoptosis in the lower hypertrophic layer of the growth plate [17]. Thus, one possibility may be that the mutant SEDLINs that are associated with SEDT and lead to a loss of interaction with MBP1 may disrupt the tight control between proliferation and apoptosis in endochondral ossification. In addition, SEDLIN has been reported to inhibit the PITX1 and SF1 mediated transactivation of the β-subunit of the luteinizing hormone [4], and the effects of the loss of interaction, due to the SEDLIN mutations, between SEDLIN and PITX1 and SF1 on the pituitary-gonadal response and the delayed puberty observed in some boys affected with SEDT [18] remain to be defined.

The Asp47Tyr SEDT-causing mutation, unlike the Ser73Leu, Phe83Ser, Val130Asp and Gln131Stop mutants, did not result in loss of interaction with MBP1, PITX1 and SF1 (Figure 7). However, the Asp47 residue, which is conserved through to yeast, has an important functional role, as in a yeast complementation study, the mutant SEDLIN, Asp47Tyr, failed to rescue the lethal *trs20p*Δ phenotype [1], [2], [19]. The basis of these major consequences of the Asp47Tyr mutation remains to be defined. However, it is interesting to note that the Asp47Tyr mutation involves a residue on the surface of SEDLIN (Figure 2) that could possibly lead to loss of postulated or known interactions with other proteins, such as: the soluble N-ethylmaleimide-sensitive factor attachment (SNARE) proteins [1], which are also involved in the vesicle transport pathway [1]; or the intracellular chloride channels CLIC1 and CLIC2 [5], respectively.

In summary, our results show that SEDLIN is a nuclear and cytoplasmic protein that forms homodimers. Moreover, 3 of the SEDLIN missense mutations (Ser73Leu, Phe83Ser and Val130Asp) and 1 nonsense mutation (Gln131Stop) located within the hydrophobic core and associated with SEDT in patients lead to a loss of interactions with the transcription factors MBP1, PITX1 and SF1.

## Materials and Methods

### Generation of SEDLIN, MBP1, PITX1 and SF1 constructs

Total RNA was isolated from HEK293 cells using Trizol (Invitrogen Corporation) and 2 µg was utilised to generate first strand cDNAs by reverse transcriptase (RT)-PCR using random hexamers and 200 U/ml Avian Myeloblastosis virus (AMV) RTase (Transgenomic Inc.) at 42°C for 45 min. The coding regions of *SEDL*, *MBP1* and *PITX1* were amplified using gene-specific primers (sequences available on request). These RT-PCR products were cloned in-frame into the expression vector pGEMT (Promega). The pGEMT-wild-type SEDLIN construct was used to generate mutant SEDLIN constructs that harboured the four missense mutations (Asp47Tyr, Ser73Leu, Phe83Ser and Val130Asp) and the C-terminal nonsense mutation Gln131Stop (Figure 1) [20]–[22] using site-directed mutagenesis (QuikChange®XL, Stratagene), as previously described [23]. The SF1 cDNA in the pCIneo vector [24] was used to subclone SF1 into pGEMT. The wild-type and mutant SEDLINs, MBP1, PITX1 and SF1 were digested from the pGEMT vector, subcloned in-frame into pCMV-cMyc, pCMV-HA, pGBKT7 and pGADT7 vectors (Clontech) and DNA sequences verified using methods previously described [23].

### Subcellular localization studies

COS7 cells, were transfected using Lipofectamine Plus (Invitrogen Corporation) with: full-length SEDLIN wild-type or mutant constructs prepared in pCMV-HA (HA-SEDLIN); and MBP1, PITX1 and SF1 prepared in pCMV-cMyc (cMyc-MBP1, cMyc-PITX1 and cMyc-SF1, respectively) as previously described [23]. Twenty hours post-transfection, cells were stained using anti-HA (Covance Inc.) and anti-cMyc (Santa Cruz Biotechnology, Inc.) primary antibodies and AlexaFluor®594 donkey anti-mouse and AlexaFluor®488 donkey anti-rabbit (Molecular Probes) secondary antibodies and visualised using a laser scanning confocal microscope (Confocal System LSM 510 META, Carl Zeiss), as previously described [25]. For Western blot analysis, cells were lysed and fractionated into nuclear and cytoplasmic extracts, and biotin-conjugated anti-HA (Vector Laboratories, Inc.) and anti-cMyc (Santa Cruz Biotechnology, Inc.) antibodies were used to detect the presence of SEDLIN, MBP1, PITX1 and SF1 proteins in the cell fractions as previously described [26]. Antibodies against Lamin A/C and α-Tubulin (Santa Cruz Biotechnology, Inc.) were used to assess the quality of the subcellular fraction preparations. Nuclear and cytoplasmic fractions (40 µg of total protein) were also analysed by 12% continuous non-denaturing PAGE in 1× Tris/Glycine buffer, transferred onto nitrocellulose membrane and hybridised with anti-cMyc antibody (Santa Cruz Biotechnology, Inc.). Secondary antibodies, horseradish peroxidase-conjugated donkey anti-goat (Santa Cruz Biotechnology, Inc.) and goat anti-mouse (Bio-Rad), were used and detected using an Enhanced Chemiluminescence (ECL) Kit (GE Healthcare).

### Co-immunoprecipitation

COS7 cells were transiently co-transfected with full-length wild-type or mutant HA-SEDLINs and cMyc-MBP1, cMyc-PITX1, cMyc-SF1, cMyc-SEDLIN or cMyc vector alone using Lipofectamine Plus (Invitrogen Corporation). After 48 hrs, cells were lysed in RIPA buffer (150 mM NaCl, 50 mM Tris-HCl pH 7.5, 1% NP-40, 0.1% SDS, 0.5% deoxycholate, 1 mM phenylmethylsulfonyl fluoride) supplemented with protease inhibitors (Complete Mini, Roche), and briefly sonicated. Co-immunoprecipitation was performed using anti-HA (1 µg, Abcam) or anti-cMyc antibodies (1 µg, Santa Cruz Biotechnology, Inc.). Protein complexes were captured with Protein G-Sepharose beads (GE Healthcare), eluted with Laemmli sample buffer (Bio-Rad) containing 5% β-mercaptoethanol and separated on a 15% discontinuous SDS-PAGE, followed by immunoblotting with biotin-conjugated anti-HA antibody (Vector Laboratories, Inc.) for the detection of HA-SEDLIN, and with anti-cMyc antibody (Santa Cruz Biotechnology, Inc.) for the detection of cMyc-MBP1, cMyc-PITX1, cMyc-SF1, or cMyc-SEDLIN proteins. Horseradish peroxidase-conjugated donkey anti-goat (Santa Cruz Biotechnology, Inc.) and goat anti-mouse (Bio-Rad), were used and detected using an Enhanced Chemiluminescence (ECL) Kit (GE Healthcare).

### Yeast two-hybrid assays

Yeast two-hybrid assays were performed as described previously [23]. Briefly, the coding regions of SEDLIN, MBP1, PITX1 and SF1 were cloned in-frame into the Gal4 activation domain encoding plasmid, pGADT7 (AD-SEDLIN, AD-MBP1, AD-PITX1 and AD-SF1), and into the Gal4 DNA-binding domain encoding plasmid, pGBKT7 (BD-SEDLIN, BD-MBP1, BD-PITX1 and BD-SF1). Mutant SEDLINs were also cloned in-frame into pGADT7 and pGBKT7. AD-MBP1, BD-PITX1 and BD-SF1 showed self-activation, whereas BD-MBP1, AD-PITX1 and AD-SF1 constructs did not self-activate, and thus these constructs were used for the study. The pGBKT7-p53 and pGADT7-Large T antigen plasmids were used as controls (Clontech) [27]. Competent AH109 yeasts cells were transformed sequentially with the appropriate wild-type and mutant SEDLIN plasmid constructs and the MBP1, PITX1 and SF1 plasmid constructs, using the LiAc/single-stranded DNA/polyethylene glycol procedure, as described previously [23]. Expression of SEDLIN, MBP1, PITX1 and SF1 fusion proteins were confirmed in protein extracts prepared from each clone, by SDS-PAGE and Western blot analysis, using anti-HA and anti-cMyc as described above.

### Computer modelling of SEDLIN structure

The three-dimensional structure of the mouse Sedlin has been reported [2], and because the mouse and human SEDLINs are 97% identical, we modelled the position of the human SEDLIN mutations on this framework. The Sedlin three-dimensional structure is archived in the Protein Data Bank (PDB) with the accession number 1H3Q [PDB] (available at www.rcsb.org/pdb/cgi/explore.cgi?pdbId=1H3Q) and was visualized using the MacPyMOL programme (DeLano Scientific LLC).
